# Structural insights into the functional roles of 14-3-3 proteins

**DOI:** 10.3389/fmolb.2022.1016071

**Published:** 2022-09-16

**Authors:** Veronika Obsilova, Tomas Obsil

**Affiliations:** ^1^ Institute of Physiology of the Czech Academy of Sciences, Laboratory of Structural Biology of Signaling Proteins, Division BIOCEV, Vestec, Czechia; ^2^ Department of Physical and Macromolecular Chemistry, Faculty of Science, Charles University, Prague, Czechia

**Keywords:** 14-3-3 proteins, protein-protein interactions, phosphorylation, molecular mechanism, scaffolding, adaptor protein

## Abstract

Signal transduction cascades efficiently transmit chemical and/or physical signals from the extracellular environment to intracellular compartments, thereby eliciting an appropriate cellular response. Most often, these signaling processes are mediated by specific protein-protein interactions involving hundreds of different receptors, enzymes, transcription factors, and signaling, adaptor and scaffolding proteins. Among them, 14-3-3 proteins are a family of highly conserved scaffolding molecules expressed in all eukaryotes, where they modulate the function of other proteins, primarily in a phosphorylation-dependent manner. Through these binding interactions, 14-3-3 proteins participate in key cellular processes, such as cell-cycle control, apoptosis, signal transduction, energy metabolism, and protein trafficking. To date, several hundreds of 14-3-3 binding partners have been identified, including protein kinases, phosphatases, receptors and transcription factors, which have been implicated in the onset of various diseases. As such, 14-3-3 proteins are promising targets for pharmaceutical interventions. However, despite intensive research into their protein-protein interactions, our understanding of the molecular mechanisms whereby 14-3-3 proteins regulate the functions of their binding partners remains insufficient. This review article provides an overview of the current state of the art of the molecular mechanisms whereby 14-3-3 proteins regulate their binding partners, focusing on recent structural studies of 14-3-3 protein complexes.

## 1 Introduction

14-3-3 proteins are a family of phosphoserine and phosphothreonine binding modules, which control almost every physiological process in the cell. Given their ubiquitous expression pattern in all tissues, they are universal scaffolds that shape the structure and function of many different protein targets and participate in all major cellular signaling pathways. All eukaryotic organisms express multiple 14-3-3 isoforms, which are encoded by different genes (van Hemert et al., 2001). Humans have seven isoforms labeled by the Greek letters *β*, *γ*, *ε*, *η*, *σ*, *τ* and *ζ* (Ichimura et al., 1988), and plants express ten different isoforms, but yeast and *Drosophila* contain only two isoforms (Aitken et al., 2002). Their peculiar name, “14-3-3”, originated from their specific elution pattern in gel-filtration fractions and their position on subsequent starch gel electrophoresis when first identified in bovine brain homogenate, in 1967 (Moore and Perez, 1967). They have since been forgotten and rediscovered again in the 1990s for their unique functions, including tyrosine and tryptophan hydroxylase (Ichimura et al., 1987), Raf-1 kinase (Fantl et al., 1994; Fu et al., 1994) and protein kinase C (Toker et al., 1990) regulations, among others. Since then, the number of binding partners has increased markedly, with a recent Gene Onthology analysis identifying well over 1,200 binding proteins in mammals, including G-proteins, many Ser/Thr protein kinases and DNA-, RNA- and calcium-binding, ubiquitination, cytoskeletal, and apoptotic proteins, among others (Sluchanko and Bustos, 2019).

Their abundant interactions reflect an extensive repertoire of cellular processes, such as transcription, intracellular targeting, cell signaling, and cell cycle control. Accordingly, 14-3-3 proteins also play a key role in a wide range of diseases. As they were originally identified in brain tissue, 14-3-3 proteins were unsurprisingly first associated with neurodegenerative diseases, including Creutzfeldt–Jakob (CJD) (Wiltfang et al., 1999), Alzheimer (Layfield et al., 1996), Parkinson (Ostrerova et al., 1999) and polyglutamine repeat (Chen et al., 2003) diseases. More recently, nevertheless, 14-3-3 proteins have also been shown to promote chemoresistance and poor outcomes among patients with cancer, such as breast, lung, prostate, head and neck cancer, as well as myeloma and glioblastoma (Lu et al., 2009; Xu et al., 2015; Gu et al., 2018; Lage-Vickers et al., 2021), most likely because the chromosomal region 8q22.3, which includes the 14-3-3ζ gene (*YWHAZ*), is regularly duplicated in cancer (Pollack et al., 2002). Therefore, in these cases, therapeutic strategies aim to disrupt 14-3-3 interactions for clinical purposes.

Since 14-3-3 protein-protein interactions (PPIs) are mediated by specific phosphopeptide motifs anchored to a well-defined ligand binding groove of the 14-3-3 molecule, they can be disrupted or stabilized by small molecules or short peptides (reviewed in (Ottmann, 2013; Stevers et al., 2018)). For example, a short peptide termed R18, which was developed by phage display, contains a negatively charged phosphorylation-mimicking sequence. This sequence, in turn, binds to the positively charged 14-3-3 ligand binding groove, thereby inhibiting 14-3-3 PPIs (Wang et al., 1999). The first proof-of-concept study targeting interactions of 14-3-3 proteins *in vivo* was reported by Fu et al., who generated difopein, a dimeric form of R18, which disrupted 14-3-3 PPIs, increased the ability of cisplatin to kill cells and triggered tumor cell apoptosis (Masters and Fu, 2001; Cao et al., 2010).

Several small-molecule 14-3-3 inhibitors have since been reported, the first of which targeted the interaction between 14-3-3 and c-Abl, thus inducing apoptosis of human leukemia cells resistant to Imatinib treatment (Corradi et al., 2010). Among other inhibitors, FOBISIN101 blocks the interaction between 14-3-3 and Raf-1 and could serve as radiation-triggered therapeutic agent (Zhao et al., 2011). Furthermore, macrocyclic peptides block the interaction between 14-3-3ζ and the virulence factor of the pathogenic bacterium *Pseudomonas aeruginosa* Exoenzyme S (ExoS) (Glas et al., 2014), and rac-UTKO1, a derivative of Moverastin, targets interactions between 14-3-3ζ and Tiam1 and βPix, thus inhibiting the migration of human esophageal tumor cells (Takemoto et al., 2005; Tashiro and Imoto, 2016).

Notwithstanding their variety, 14-3-3 PPI inhibitors still lack specificity to 14-3-3 isoforms because these compounds often target the ligand binding groove, which displays the highest sequence homology among the isoforms. However, the isoforms tend to compensate for each other, as previously demonstrated (Acevedo et al., 2007; Gogl et al., 2021). Hence, targeting multiple 14-3-3 isoforms could be beneficial under such conditions.

Among the 14-3-3 isoforms, *σ* stands out as a functional exception because this isoform serves as a tumor suppressor by positively regulating p53 (Yang et al., 2003). In addition, its expression is also downregulated in breast cancer (Ferguson et al., 2000), but most cancers have high levels of 14-3-3ζ. Consequently, identifying small-molecule inhibitors of this isoform could foster the development of treatments for a number of cancers.

In many other situations, however, stabilizing 14-3-3 PPIs could also be beneficial. Oecking and colleagues were the first to demonstrate that 14-3-3 PPIs can be stabilized by targeting one side of the 14-3-3 ligand binding groove using the diterpene glycoside toxin fusicoccin A (FC-A) produced by the phytopathogenic fungus *Phomopsis amygdali* (Oecking et al., 1994; Wurtele et al., 2003; Ottmann et al., 2007a). Their studies showed that fusicoccin A fills a gap in the interface between the 14-3-3 ligand-binding groove and the phosphopeptide, thereby enhancing its binding.

Since then, many other 14-3-3 PPI stabilizers have been reported including cotylenin-A (Molzan et al., 2013), pyrrolidone 1, epibestatin (Rose et al., 2010), adenosine monophosphate (Sato et al., 2016), phosphonate derivatives (Sijbesma et al., 2020) and macrocyclic compounds (Stevers et al., 2022), among others. These structural and functional findings highlighted the potential of 14-3-3 PPI stabilization as a promising option in drug discovery. For example, 14-3-3 proteins are positive regulators of the chloride channel cystic fibrosis transmembrane conductance regulator (CFTR), whose F508del mutation is frequent cause of cystic fibrosis (Lukacs et al., 1993). The Ottmann group demonstrated the druggability of the 14-3-3:CFTR interface (Stevers et al., 2016), in addition to showing that stabilizing this complex with macrocyclic compounds rescues plasma membrane localization and chloride transport of CFTR F508del. Combined, these results support the use of these compounds in the treatment of cystic fibrosis (Stevers et al., 2022).

Considering the above, the aim of this review article is to provide an overview of our current knowledge on structural studies of 14-3-3 protein complexes and mechanisms of function of 14-3-3 proteins in the regulation of their binding partners. For further information, the reader is also invited to peruse several other excellent reviews on 14-3-3 proteins and their functions (Fu et al., 2000; Bridges and Moorhead, 2004; Mackintosh, 2004; Pennington et al., 2018; Sluchanko, 2022).

## 2 Structure of 14-3-3 proteins and their binding motifs

### 2.1 Structure of 14-3-3 proteins

**FIGURE 1:**
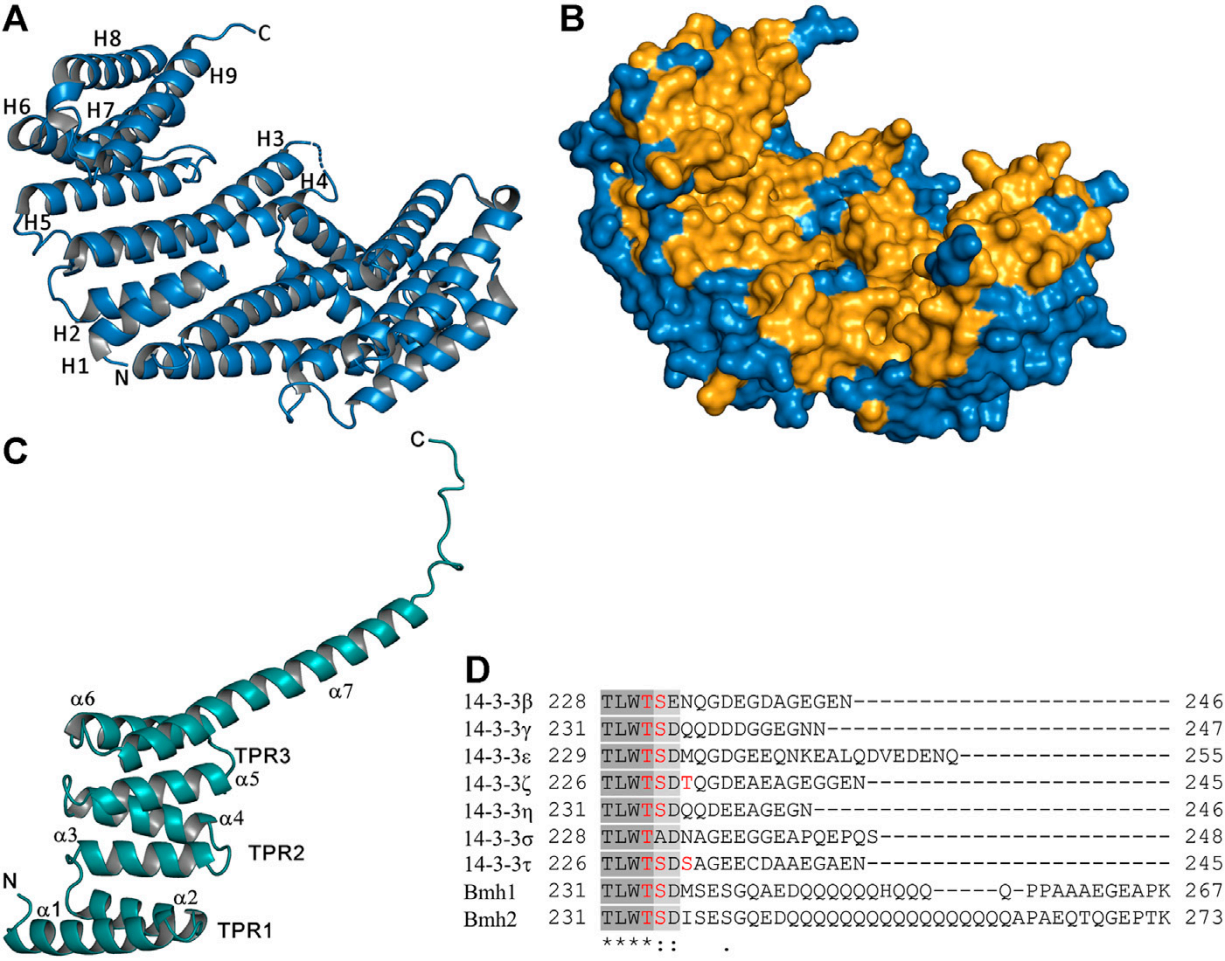
Structure of 14-3-3 proteins. **(A)** Crystal structure of the human 14-3-3 protein isoform *ζ*, the ribbon representation, and *α*-helices of one protomer are labeled (PDB ID: 1QJB) (Rittinger et al., 1999). **(B)** Surface representation of the human 14-3-3 protein isoform *ζ*; the totally conserved residues among all seven human and two yeast 14-3-3 isoforms are shaded in bright orange. **(C)** Crystal structure of the TPR domain of PP5, *α*-helices and TPR motifs formed by two α-helices are labeled (PDB ID: 1A17) (Das et al., 1998). **(D)** Sequence alignment of the C-terminal part of the seven human (*β*, *γ*, *ε*, *ζ*, *η*, *σ* and *τ*) and two yeast (Bmh1 and Bmh2) 14-3-3 protein isoforms using Clustal Omega; phosphorylation sites present in this region are highlighted in red (Pennington et al., 2018). The figure was prepared with PyMOL (https://pymol.org/2/).

The crystal structures of all seven human isoforms of 14-3-3 proteins have already been reported, starting with isoforms τ and ζ (Liu et al., 1995; Xiao et al., 1995; Yang et al., 2006). These structures revealed the dimeric nature of 14-3-3 proteins and their high helical content (Figures 1A,B). Each protomer with a molecular mass of ∼30 kDa consists of nine antiparallel α-helices (H1-H9) and contains one phosphopeptide binding site. The dimer forms a cup-shaped particle, with twofold symmetry, which has a central channel formed by helices H3, H5, H7 and H9 with the following dimensions: 35 Å × 35 Å × 20 Å (length × width × height). Moreover, this central channel contains two ligand binding grooves. The residues that form the inner surface of the dimer that includes both ligand binding grooves are highly conserved, whereas the residues that form the surface outside the central channel exhibit high variability. 14-3-3 proteins have a rather distinct domain structure, which presumably evolved from tetratricopeptide repeat (TPR) proteins (Zhu et al., 2016). The TPR repeat is a 34-amino-acid-long structural motif present in tandem arrays of 3–16 motifs, thus forming scaffolds able to mediate protein-protein interactions in the same manner as 14-3-3 proteins (Figure 1C) (Das et al., 1998).

### 2.2 Structural differences among 14-3-3 isoforms

Some 14-3-3 ligand proteins appear to have a distinct preference for specific 14-3-3 isoforms. A typical example is an isoform-specific interaction between 14-3-3*γ* and the ERK1/2 scaffold KSR1, which protects KSR1 from epidermal growth factor-induced dephosphorylation, blocking its ability to activate ERK2 (Jagemann et al., 2008). However, the exact nature of such isoform-specific interactions remains largely unknown. Structural comparisons of crystal structures of human 14-3-3 protein isoforms have shown that they are similar, but the individual structures exhibit slight differences in the angle between the two subunits, in the length of loop regions and in the length of α-helices, especially helices H3 and H4 and the loop between them (Gardino et al., 2006; Yang et al., 2006). The loops connecting helices H3-H4 and H8-H9 are often disordered in available crystal structures, indicating their high flexibility.

The largest sequence variability between 14-3-3 protein isoforms occurs in the C-terminal stretch, which is disordered and thus not visible in crystal structures (Figure 1D). This flexible region contains 15–40 amino acids, including negatively charged Glu and Asp residues, which can interact with the positively charged phospho-binding pocket of 14-3-3. Through these interactions, the C-terminal stretch acts, at least in some isoforms, as a 14-3-3 auto-inhibitor regulating binding properties by blocking non-specific interactions (Truong et al., 2002; Obsilova et al., 2004). We have also shown that, in the absence of a ligand, the C-terminus of 14-3-3ζ is located in the ligand binding groove and that phosphopeptide binding displaces the C-terminal stretch from the groove, adopting a new conformation above the helix H9 (Silhan et al., 2004). In addition, the C-terminal stretch contains a cluster of several phosphosites of unknown function, which could further modulate the binding properties of 14-3-3 isoforms (Figure 1D) (Obsilova et al., 2004; Pennington et al., 2018).

In contrast to mammalian 14-3-3 isoforms, the yeast homologs Bmh1 and Bmh2 proteins have a noticeably different C-terminal tail. Their C-terminus is not only longer but also contains a polyglutamine sequence, with an unknown function, and adopts a widely opened and extended conformation, which prevents its folding into the ligand binding groove. Therefore, unlike in human 14-3-3ζ, in Bmh1 and Bmh2, the C-terminus does not function as an auto-inhibitor (Veisova et al., 2010).

### 2.3 Homo- and heterodimerization of 14-3-3 proteins

The scaffolding function of 14-3-3 proteins is closely related to their dimeric form, which is stabilized by numerous hydrophobic and electrostatic interactions at the interface between protomers, as reviewed in (Sluchanko and Gusev, 2012). The sequences of individual 14-3-3 isoforms differ in the regions of inter-subunit contacts. Thus, the homodimers of different 14-3-3 isoforms are stabilized by a different number of salt bridges, namely one in 14-3-3*ε*, two in 14-3-3*γ* and *η*, and three in 14-3-3β, *ζ*, *σ* and *τ* (Gardino et al., 2006). Moreover, the dimeric structure of 14-3-3 can be stabilized by alternative salt bridges not observed in the crystal structure, as identified in 14-3-3ζ (Haladova et al., 2012).

Individual 14-3-3 isoforms also differ in their ability to form heterodimers (Jones et al., 1995). For example, 14-3-3σ forms exclusively homodimers (Verdoodt et al., 2006), 14-3-3ζ forms both homo- and heterodimers with five other isoforms, and 14-3-3ε preferentially forms heterodimers stabilized by more than one salt bridge (Yang et al., 2006). Because 14-3-3 isoforms often differ in binding affinities to their targets (Gogl et al., 2021), the formation of different 14-3-3 homo- and heterodimers may play a key role in signaling pathways, especially when the target protein contains two 14-3-3 binding sites or when the 14-3-3 dimer simultaneously interacts with two different binding partners. In addition, several 14-3-3 binding partners selectively interact with specific 14-3-3 heterodimers. For instance, the 14-3-3β/ε heterodimer is required for aldosterone regulation of the epithelial sodium channel (ENaC) (Liang et al., 2008), whereas the 14-3-3ζ/τ heterodimer regulates the activity of Slingshot phosphatase in keratinocytes (Kligys et al., 2009).

The dimerization of 14-3-3 proteins is controlled by post-translational modifications. Thus far, the most studied post-translational modification is the phosphorylation of Ser^58^, located at the dimer interface, which is mediated by several kinases, such as PKA, Akt/PKB, PKCδ or SDK, usually under stress conditions (Powell et al., 2002; Woodcock et al., 2003; Gu et al., 2006; Gerst et al., 2015). Ser^58^ phosphorylation has been shown to severely impact the dimerization of 14-3-3 proteins (Woodcock et al., 2003; Zhou et al., 2009; Kanno and Nishizaki, 2011), as recently confirmed and quantitatively described by the Hritz group, who determined that the *K*_*D*_ value of 14-3-3ζ homodimerization (ζ/ζ) is ∼5 nM using various biophysical approaches (Trosanova et al., 2022). In contrast, 14-3-3ζ phosphorylation at Ser^58^ (pζ) decreases this value to ∼4 mM (pζ/pζ), demonstrating the strong effect of Ser^58^ phosphorylation on 14-3-3ζ dimerization. In turn, the *K*_*D*_ value of phosphorylated and non-phosphorylated 14-3-3ζ (pζ/ζ) heterodimerization is ∼3 μM. Kinetic measurements also revealed that the lifetime of the 14-3-3ζ homodimer is 6 min at 37°C, thus matching the dissociation rate constant (*k*_off_) of ∼3 × 10^–3^ s^−1^.14-3-3ζ is the most abundantly expressed isoform in the human brain, with a concentration of approximately 100 μM. Based on these results, the concentration of monomeric 14-3-3ζ in brain tissue is ∼0.5 μM, with the lifetime of 4 s (Trosanova et al., 2022).

### 2.4 Canonical 14-3-3 binding motifs and their recognition

**FIGURE 2:**
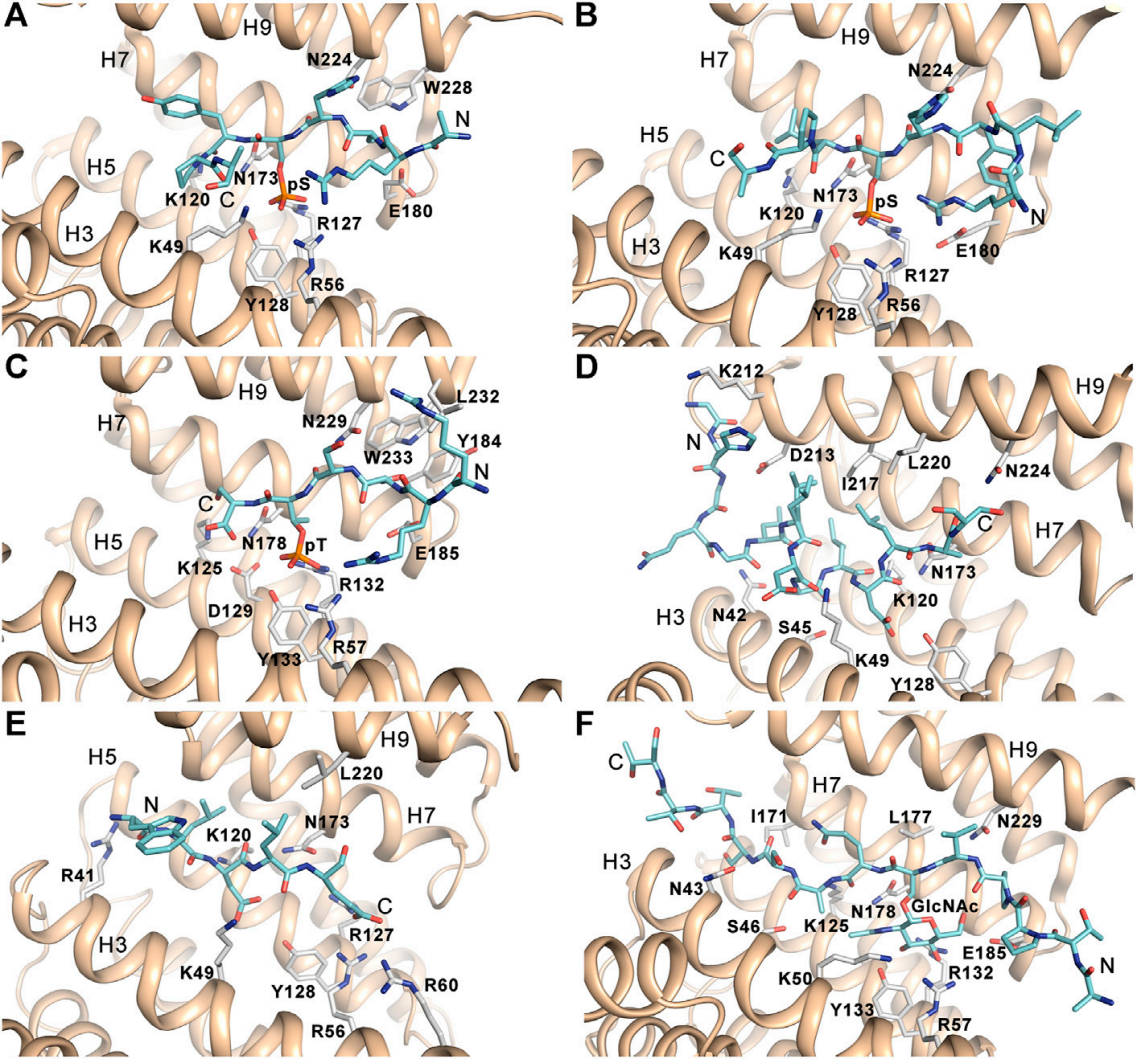
14-3-3 binding motifs. **(A)** The canonical mode I phosphopeptide (sequence ARSH(pS)YPA) bound to 14-3-3ζ (PDB ID: 1QJB) (Rittinger et al., 1999). **(B)** The canonical mode II phosphopeptide (sequence RLYH(pS)LPA) bound to 14-3-3ζ (PDB ID: 1QJA) (Rittinger et al., 1999). **(C)** The canonical mode III phosphopeptide derived from the C-terminus of human DAPK2 (sequence RRSS(pT)S) bound to 14-3-3γ (PDB ID: 7A6R) (Horvath et al., 2021). **(D)** The non-phosphorylated ExoS peptide (sequence GHGQGLLDALDLAS) bound to 14-3-3ζ (PDB ID: 2O02) (Ottmann et al., 2007b). **(E)** The non-phosphorylated R18 peptide derived from phage display (sequence WLDLE) bound to 14-3-3ζ (PDB ID: 1A38) (Petosa et al., 1998). **(F)** The glycosylated Ser-O-GlcNAc peptide (sequence ATPPV(S-O-GlcNAc)QASSTTT) bound to 14-3-3γ (PDB ID: 6BYJ) (Toleman et al., 2018). The figure was prepared with PyMOL (https://pymol.org/2/).

14-3-3 proteins specifically recognize phosphoserine- or phosphothreonine-containing motifs (Muslin et al., 1996). Two optimal binding motifs were initially identified when screening an oriented peptide library, namely R [S/Φ][+](pS/pT)XP (mode I) and RX [Φ/S][+](pS/pT)XP (mode II), where pS/pT is phosphoserine or phosphothreonine, Φ is an aromatic residue, + is a basic residue, and X is any type of residue (typically Leu, Glu, Ala, and Met) (Figures 2A,B) (Yaffe et al., 1997; Rittinger et al., 1999). More recently, a third canonical binding motif (pS/pT)X_1–2_-COOH) was identified, leaving a most of the 14-3-3 binding groove empty (Figure 2C) (Ganguly et al., 2005). These three motifs, albeit optimal, are not absolute because many 14-3-3 binding partners contain considerably different motifs (Johnson et al., 2010).

Dozens of structures of 14-3-3 protein complexes with short synthetic phosphopeptides currently available have shown that phosphopeptide binding does not induce substantial changes in the structure of the 14–3-3 dimer (Yaffe et al., 1997; Rittinger et al., 1999; Wurtele et al., 2003; Yang et al., 2006; Ottmann et al., 2007b; Sluchanko et al., 2017; Psenakova et al., 2018; Pohl et al., 2021). The phosphate group of pSer/pThr is recognized by ionic and hydrogen bonds to conserved residues Lys^49^, Arg^56^, Arg^127^ and Tyr^128^ (human isoform ζ numbering) located in helices H3 and H5, which form a positively charged pocket within the amphipatic ligand binding groove of 14-3-3 (Figures 2A–C). Due to interactions between the phosphopeptide backbone and side chains of conserved residues Asn^173^ and Asn^224^ and to the presence of a conserved hydrophobic patch, the bound phosphopeptides adopt an extended conformation with a fixed orientation in the binding groove. The typical preference for a basic residue (Arg or Lys) at position -3 with respect to the phosphoresidue results from contacts with acidic residues in its vicinity. Moreover, the Pro residue often found at position +2 in 14-3-3 binding motifs allows an abrupt change in the peptide main chain direction and its exit from the ligand binding groove.

### 2.5 Not-phosphorylated and novel 14-3-3 binding motifs

Although most binding partners of 14-3-3 proteins contain phosphorylated binding motifs, 14-3-3 proteins can also bind to non-phosphorylated motifs with high affinity. The first phosphorylation-independent interaction of 14-3-3 with a ligand was identified for exoenzyme S (ExoS), a bacterial ADP-ribosyltransferase toxin in *Pseudomonas aeruginosa* (Fu et al., 1993; Masters et al., 1999). This interaction is essential for toxin function, and Leu^428^ is the most critical residue, as shown by mutagenesis and cytotoxicity analysis, whereby substitution of this leucine attenuated the ability of ExoS to mediate cell death. The structure of the complex between 14-3-3ζ and the ExoS peptide containing the amphipathic motif L^426^DLA^429^ (LDLA-box) reported by Ottmann et al. revealed that the ExoS peptide binds to 14-3-3 in the reverse orientation, mainly through hydrophobic contacts, with electrostatic interactions playing only a marginal role (Figure 2D) (Ottmann et al., 2007b).

Another well-characterized peptide to which 14-3-3 proteins bind with high affinity is the aforementioned R18 peptide derived from the phage display library (Wang et al., 1999). The comparison between the crystal structures of 14-3-3ζ with the c-Raf-derived phosphopeptide and the R18 peptide revealed that R18 binds to14-3-3ζ similarly to phosphopeptides through the amphipathic WLDLE motif. The two acidic residues of this motif coordinate the same cluster of basic residues within the ligand binding groove (Figure 2E) (Petosa et al., 1998).

Interestingly, 14-3-3 proteins have also been shown to selectively bind to motifs containing an O-linked β-N-acetylglucosamine (O-GlcNAc) moiety, which is a common reversible posttranslational modification of serine and threonine residues in nuclear and cytoplasmic proteins, both in animals and plants (Toleman et al., 2018). This finding not only further expands the repertoire of 14-3-3 PPIs but may also enable crosstalk between the O-GlcNAc and O-phosphate signaling pathways. The authors also reported crystal structures of 14-3-3β and ζ proteins bound to glycopeptide, which revealed that the glycopeptide occupies the same ligand binding groove as the phosphorylated motif, making similar contacts (Figure 2F).

## 3 Functional roles of 14-3-3 proteins

In addition to the high number of structures of 14-3-3 protein complexes with short phosphopeptides, several structures of 14-3-3 protein complexes with complete binding partners are also available. Thanks to these structures, we now have a detailed understanding of the mechanisms through which 14-3-3 proteins regulate their binding partners. These mechanisms can be divided into three categories: 1) direct conformational modulation of the bound partner; 2) physical occlusion of sequence-specific or structural features on the surface of the target protein; and 3) scaffolding, which facilitates protein-protein interactions. Furthermore, in some cases, multiple mechanisms are simultaneously used to modulate the function of the target protein.

### 3.1 Allosteric regulation of enzymatic activity

14-3-3 proteins interact with many enzymes, such as arylalkylamine N-acetyltransferase (AANAT), tyrosin hydroxylase, tryptophan hydroxylase, yeast neutral trehalase (*N*th1), apoptosis signal-regulating kinases (ASK1/2), protein kinases B-RAF and C-RAF, leucine-rich repeat protein kinase-2 (LRRK2), protein kinase C (PKC), calcium/calmodulin-dependent protein kinase kinases (CaMKK1/2), death-associated protein kinase 2 (DAPK2), phosphatidylinositol-4-kinase-III (PI4KB), protein phosphatase CDC25C, and E3 ligase neural precursor cell expressed developmentally down-regulated 4 ligase (Nedd4-2), among others. The activity of these enzymes is modulated by 14-3-3 proteins through various mechanisms, several of which are based on direct conformational changes, as shown in AANAT, *N*th1 and Nedd4-2. X-ray structural analysis of AANAT and *N*th1 and a hybrid approach to the structural characterization of Nedd4-2, combining SAXS, chemical cross-linking and fluorescence spectroscopy, have enabled a detailed understanding of how 14-3-3 proteins regulate these enzymes, as discussed below.

#### 3.1.1 Arylalkylamine N-acetyltransferase (AANAT) regulation

**FIGURE 3:**
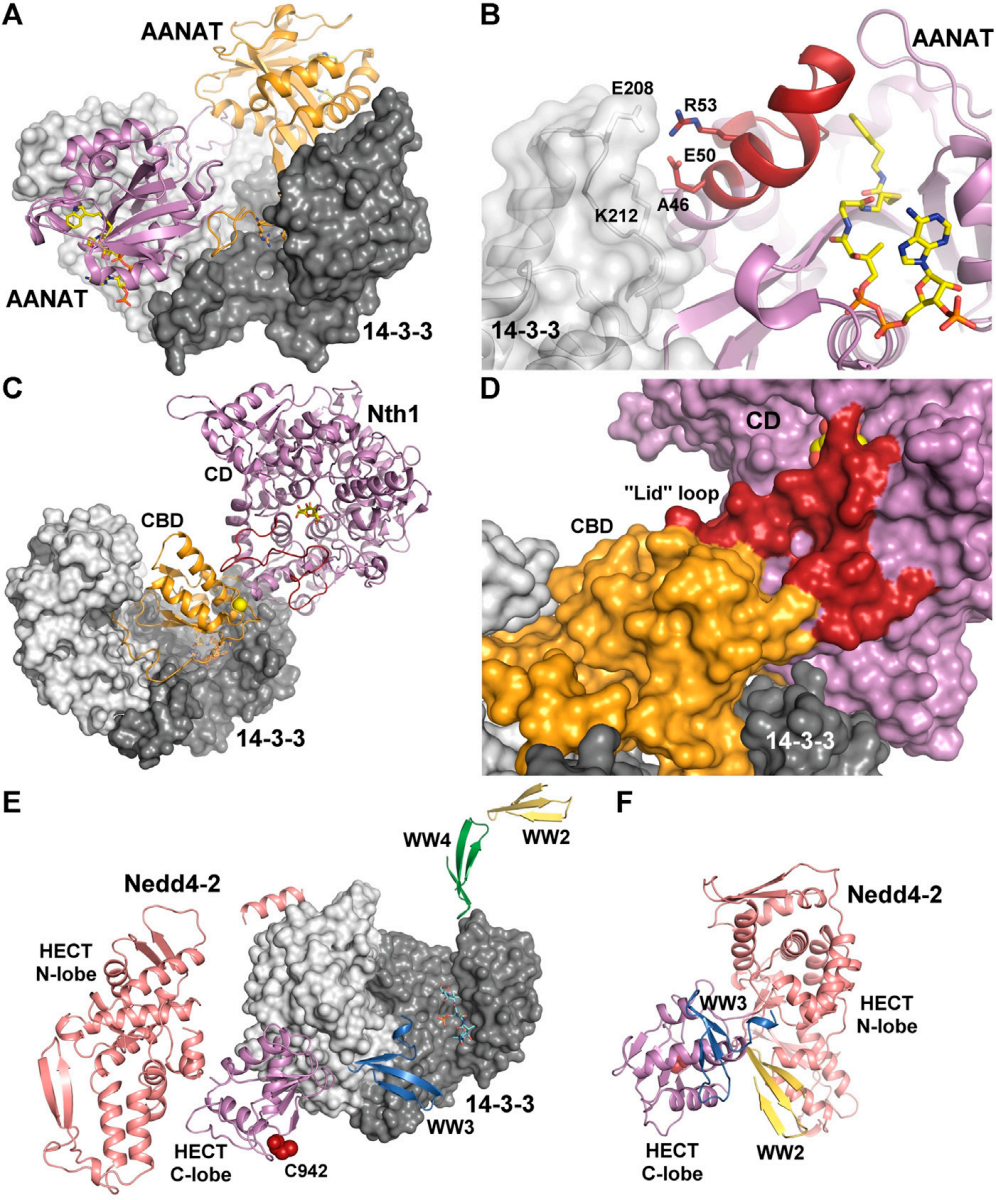
14-3-3 proteins as allosteric modulators of bound enzymes. **(A)** Crystal structure of the AANAT:14-3-3ζ complex (PDB: 1IB1 (Obsil et al., 2001)). The bisubstrate analog in shown as sticks. **(B)** Detailed view of interactions between the active site of AANAT and 14-3-3ζ. The bisubstrate analog and selected residues at the binding interface are shown as sticks. The region that undergoes a large structural rearrangement upon substrate binding is shown in dark red (Hickman et al., 1999a; Hickman et al., 1999b). **(C)** Crystal structure of the *N*th1:14-3-3 complex (PDB: 5N6N (Alblova et al., 2017)). The Ca^2+^ -binding domain (CBD) is shown in orange, the catalytic domain (CD) is shown in violet. The flexible part of the active site, which contains residues required for catalysis and is stabilized upon formation of the complex (the “lid” loop) is shown in dark red. The sucrose bound in the active site and 14-3-3 binding motifs are shown as sticks. Ca^2+^ ion is shown as yellow sphere. **(D)** Detailed view of the “lid” loop in surface representation. 14-3-3 binding allows proper orientation of the regulatory CBD of *N*th1 relative to the CD, thereby stabilizing the “lid” loop. **(E)** SAXS- and chemical cross-linking-based model of the Nedd4-2:14-3-3η complex (Pohl et al., 2021). The N- and C-lobes of the catalytic HECT domain of Nedd4-2 are shown in salmon and violet, respectively. WW2, WW3, and WW4 domains are indicated in yellow, blue and green, respectively. The catalytic Cys^942^ is shown in dark red. The 14-3-3 binding motifs are shown as sticks. **(F)** SAXS- and chemical cross-linking-based model of apo Nedd4-2. All colors are the same as in panel **(E)**. The figure was prepared with PyMOL (https://pymol.org/2/).

AANAT is the penultimate enzyme in the biosynthesis of melatonin. Its phosphorylation by the cAMP-dependent protein kinase (PKA) at Thr^31^ and Ser^205^ generates two 14–3-3 binding motifs, which border the catalytic domain. The formation of the AANAT:14-3-3ζ complex substantially increases the affinity of the enzyme to its substrates, thus enhancing melatonin production at low substrate concentrations (Ganguly et al., 2001). The crystal structure of the AANAT:14-3-3ζ complex revealed that, in addition to contacts in the phosphopeptide binding groove of 14-3-3ζ, the 14-3-3ζ loop connecting helices H8 and H9 interacts with the helix α1 of AANAT (Figures 3A,B) (Obsil et al., 2001). This region of AANAT forms one side of the active site and undergoes a large structural rearrangement upon substrate binding (Hickman et al., 1999a; Hickman et al., 1999b). The structure of the complex, together with isothermal titration calorimetry measurements, suggested that 14-3-3ζ binding stabilizes this part of the active site and forces AANAT to adopt a conformation that allows optimal substrate binding.

#### 3.1.2 Yeast neutral trehalase *N*th1 regulation

Another well-characterized example of 14-3-3-mediated allosteric regulation is the activation of *Saccharomyces cerevisiae* neutral trehalase *N*th1. *N*th1 catalyzes the hydrolysis of the nonreducing disaccharide trehalose (α-d-glucopyranosyl α-d-glucopyranoside), and its activity is triggered by PKA-mediated phosphorylation of two 14–3-3 binding motifs in the N-terminal region of the enzyme (Ser^60^ and Ser^83^), followed by binding to yeast 14-3-3 proteins (Bmh) (Panni et al., 2008; Veisova et al., 2012; Macakova et al., 2013). In yeast, *N*th1 activation is essential for energy metabolism and cell cycle progression because the intracellular trehalose levels are under strong cell cycle control (Ewald et al., 2016). Structural and biophysical analyses have revealed that 14–3-3 binding stabilizes the regulatory Ca^2+^-binding domain of *N*th1 and allows its proper orientation, relative to the catalytic domain, thereby stabilizing the flexible part of the active site, which contains residues required for catalysis (Figures 3C,D) (Kopecka et al., 2014; Alblova et al., 2017). The crystal structure of the *N*th1:14-3-3 complex, the first example of a 14-3-3 complex with a multidomain partner, demonstrated the ability of 14-3-3 proteins to modulate interdomain interactions in such binding partners.

#### 3.1.3 E3 ligase Nedd4-2 regulation

The interaction between E3 ligase Nedd4-2 and 14-3-3 proteins is another example of 14-3-3-dependent regulation of a multidomain enzyme. Nedd4-2 has three distinct types of domains, namely the N-terminal C2 domain, four WW domains and the HECT domain. Nedd4-2 activity is negatively regulated by phosphorylation mediated by various kinases, such as IKKβ, Akt/PKB, SGK1 and PKA, leading to 14-3-3 protein binding, thereby inhibiting Nedd4-2 binding to ENaC (Bhalla et al., 2005; Ichimura et al., 2005; Lee et al., 2007; Liang et al., 2008; Edinger et al., 2009). Although calcium has been shown to markedly increase the ubiquitin ligase activity of Nedd4-2 in a process requiring N-terminal C2 domain binding to the membrane to stabilize the active conformation, 14-3-3 binding inhibits Nedd4-2 activity. Recently, we and others have shown that the key sites responsible for the interaction with 14-3-3 are Ser^342^ and Ser^448^ phosphorylation sites, located near the WW2 domain and that Ser^448^ is the dominant site, resembling the mode I consensus motif (Chandran et al., 2011; Pohl et al., 2021). SAXS-based structural analysis combined with chemical cross-linking and fluorescence spectroscopy provided the first glimpse into the 14-3-3-mediated inhibition of Nedd4-2, showing that 14-3-3 binding induces a structural rearrangement of Nedd4-2 by altering interactions between its structured domains (Figures 3E,F) (Pohl et al., 2021). Furthermore, the formation of the complex causes both steric hindrance of the WW3 and WW4 domains and a conformational change of the catalytic HECT domain (Joshi et al., 2022). Because WW domains presumably mediate Nedd4-2 binding to its substrates, such occlusions combined with conformational changes in the catalytic domain likely affect substrate ubiquitination, thus explaining the 14-3-3-mediated modulation of the ubiquitination of some Nedd4-2 substrates.

### 3.2 Mechanisms based on physical occlusion of sequence-specific or structural features

#### 3.2.1 Regulation of subcellular localization

14-3-3 protein binding motifs are often located near the nuclear localization sequence (NLS) or nuclear export sequence (NES). If so, 14-3-3 binding can modulate the subcellular localization of its target by physically masking its NLS or NES (reviewed in (Muslin and Xing, 2000)). A well-known example of such a mode of regulation involves Forkhead box O transcription factors (FOXO), which are crucial for cell survival, DNA damage repair, and stress resistance (reviewed in (Gui and Burgering, 2021)). Their transcriptional activity is regulated by the insulin/IGF-1 signaling pathway through phosphorylation of three conserved Ser/Thr residues by Akt/PKB kinase, two of which are 14-3-3 binding motifs (Thr^32^ and Ser^197^ in human FOXO4). 14-3-3 protein binding to phosphorylated FOXOs induces their rapid translocation to the cytoplasm, thereby inhibiting their transcriptional activity (Brunet et al., 1999; Brunet et al., 2002). The NLS of FOXO proteins is located close to the second 14-3-3 binding motif at the C-terminus of the Forkhead domain, suggesting that 14-3-3 binding could interfere with the nuclear localization function of the NLS (Brunet et al., 2002; Zhao et al., 2004). Indeed, our biophysical study confirmed that 14-3-3ζ protein directly interacts with the NLS of FOXO4, thus supporting this hypothesis (Obsilova et al., 2005).

Another example of this regulatory mechanism is the 14-3-3-mediated inhibition of the nuclear import of dual specificity phosphatases Cdc25B and Cdc25C. Cdc25B, which is involved in the G2/M transition of the cell cycle, contains a 14-3-3-binding site (Ser^323^) adjacent to the NLS. After its phosphorylation and 14-3-3 binding, the CDC25B:14-3-3 complex is shuttled into the cytoplasm (Davezac et al., 2000). Cdc25C shuttling mirrors this mechanism (Graves et al., 2001; Margolis et al., 2006). In another mechanism of 14-3-3-mediated regulation of subcellular localization, the proapoptotic protein BAD promotes cell death when in a complex with Bcl-x_L_ localized in the mitochondrial membrane. However, BAD phosphorylation at Ser^136^ recruits 14-3-3 proteins, whose binding allows the phosphorylation of another site, Ser^155^, thereby disrupting the BAD:Bcl-x_L_ complex and sequestering the BAD:14-3-3 complex in the cytoplasm (Zha et al., 1996; Datta et al., 2000; Tan et al., 2000).

The subcellular localization of class II histone deacetylases (HDAC4 and HDAC5) is also regulated in a phosphorylation- and 14-3-3-dependent manner. HDAC4 and HDAC5 contain two 14-3-3 binding motifs, a NLS located between these two motifs, and a NES at the C-terminus, which is inactive in unphosphorylated HDACs. The current model for signal-dependent nuclear export of HDACs assumes that their phosphorylation in response to CaMK signaling and subsequent binding to 14-3-3 proteins expose the C-terminal NES and simultaneously mask the NLS, thereby inducing HDACs sequestration in the cytoplasm (Grozinger and Schreiber, 2000; McKinsey et al., 2000, 2001). Another protein whose NLS is masked by 14-3-3 binding is caspase-2, an apical protease responsible for the proteolysis of cellular substrates involved in apoptotic signaling cascades. Procaspase-2 (an immature form of caspase-2) activation is inhibited by phosphorylation and subsequent binding to 14-3-3 proteins (Nutt et al., 2009). Structural characterization of the procaspase-2:14-3-3ζ complex has indicated that 14-3-3 binding may regulate caspase-2 activation through two distinct processes, namely interference with procaspase-2 oligomerization by masking its p12 domain and/or its nuclear localization by blocking the NLS, which is located between two 14-3-3-binding motifs of procaspase-2 (Kalabova et al., 2017; Smidova et al., 2018; Kalabova et al., 2020). However, whether NLS masking leads to procaspase-2 sequestration in the cytoplasm has not been shown yet.

14-3-3 proteins could also induce nuclear localization or the synaptic localization and surface delivery of their targets. For example, 14-3-3 binding enhances human telomerase (TERT) nuclear localization by blocking the interaction between the NES-like motif of TERT and the receptor for the nuclear export machinery (CRM1/exportin 1) (Seimiya et al., 2000). 14-3-3 proteins also positively regulate long-term potentiation learning and memory by promoting the synaptic localization and surface delivery of N-methyl-d-aspartate receptors (NMDARs) (Qiao et al., 2014; Lee et al., 2021). These studies have suggested that stabilizing the interaction between NMDARs and 14-3-3 proteins may be an efficient therapeutic approach because NMDAR hypofunction triggers symptoms of schizophrenia and its associated behaviors (reviewed in (Nakazawa et al., 2017)).

#### 3.2.2 Protection against dephosphorylation

Another regulatory mechanism based on molecular interference is the protection of phosphorylated motifs from dephosphorylation, which applies to not only 14-3-3 binding motifs but also phosphomotifs located near the binding interface. For example, Ca^2+^/calmodulin-dependent protein kinase kinases (CaMKKs) 1 and 2 are involved in adiposity regulation, glucose homeostasis and cancer, and their activity is inhibited by phosphorylation followed by association with 14-3-3 proteins (Soderling, 1999; Davare et al., 2004). We and others have shown that 14-3-3 proteins contribute to CaMKKs inhibition by protecting the inhibitory phosphorylation sites that are not 14-3-3 binding motifs from dephosphorylation (shown for both CaMKK1 and CaMKK2), in addition to direct inhibition (observed only in CaMKK1), which may be at least partly due to Ca^2+^/CaM binding suppression (Davare et al., 2004; Ichimura et al., 2008; Psenakova et al., 2018).

A similar mechanism also contributes to 14-3-3-dependent inhibition of CaM-regulated Ser/Thr protein kinase DAPK2, which is involved in apoptosis, autophagy, granulocyte differentiation and motility regulation (reviewed in (Bialik and Kimchi, 2006)). DAPK2 kinase activity is suppressed through autoinhibition, homodimerization and 14-3-3 binding to the C-terminal canonical mode III phosphorylated motif (Gilad et al., 2014; Yuasa et al., 2015). Our recent biophysical characterization of the interaction between autophosphorylated DAPK2 and 14-3-3γ has revealed that the formation of the complex stabilizes DAPK2 dimerization, protects the DAPK2 inhibitory autophosphorylation site Ser^318^ against dephosphorylation and suppresses Ca^2+^/CaM binding (Horvath et al., 2021). Therefore, both CaMKKs and DAPK2 are paradigmatic examples of target proteins regulated by 14-3-3 through several mechanisms.

#### 3.2.3 Chaperon-like activity and interference with protein-protein and protein-DNA interactions

In addition to phosphorylation-specific interactions, 14-3-3 proteins also exhibit phosphorylation-independent chaperone-like activity (reviewed in (Sluchanko and Gusev, 2017)). This activity prevents aggregation of partly folded or misfolded proteins and is both ATP-independent and higher in monomeric forms of 14-3-3 proteins, presumably due to the higher exposure of hydrophobic residues (Sluchanko et al., 2011). Furthermore, 14-3-3 proteins are also closely involved in the regulation of small heat shock proteins. For instance, 14-3-3 proteins interact with the phosphorylated form of HSPB6 and stabilize its intrinsically disordered N-terminal domain (Sluchanko et al., 2017). This interaction seems to be essential to the regulation of smooth muscle contraction, most likely by turning phosphorylated HSPB6 into a competitor for 14-3-3 binding (Dreiza et al., 2005).

**FIGURE 4:**
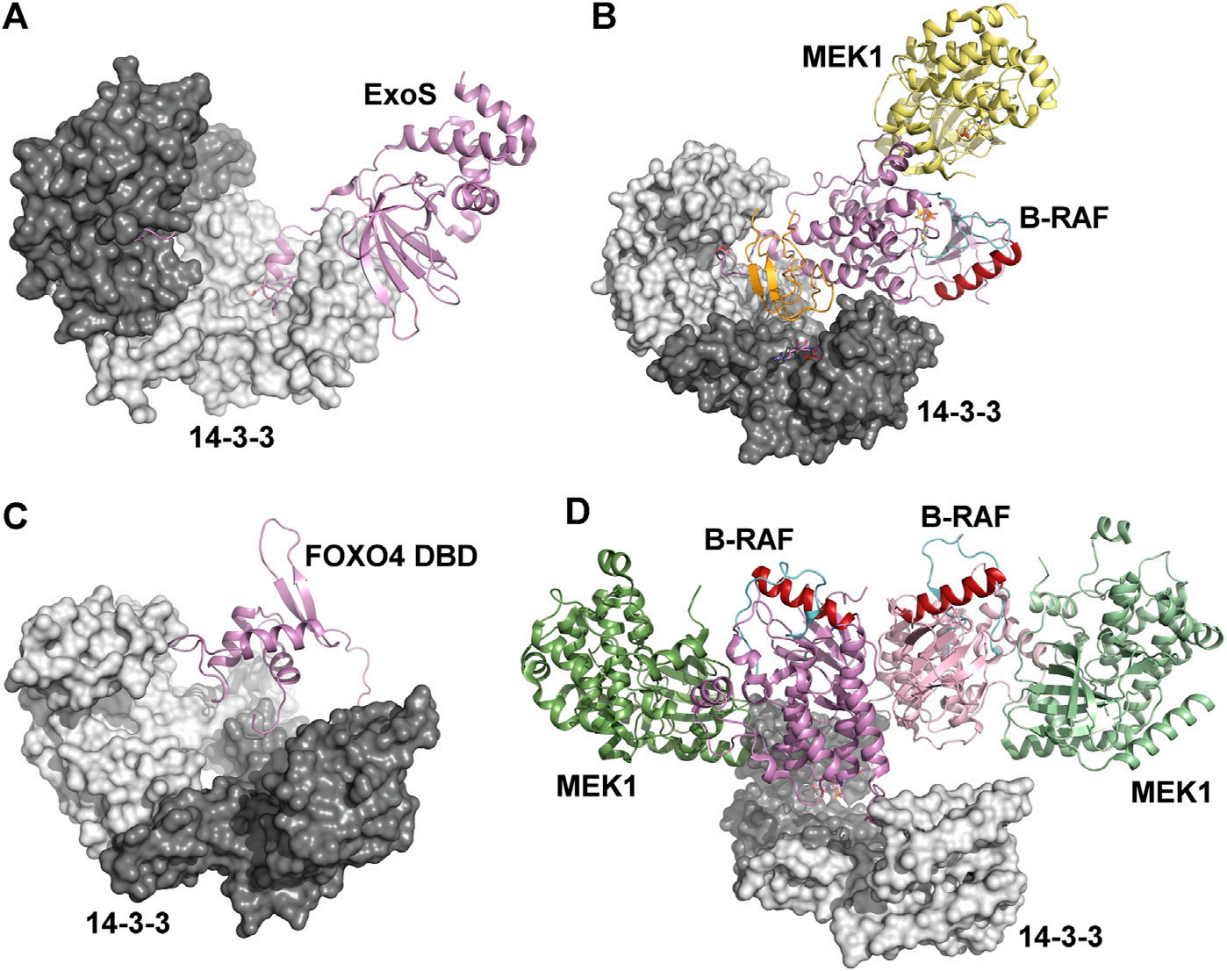
Mechanisms based on physical occlusion and facilitation of protein-protein interactions. **(A)** Crystal structures of the ExoS:14-3-3β complex showing an extensive hydrophobic binding interface. The amphipathic 14-3-3 binding LDLA-motif is shown as sticks (Karlberg et al., 2018). **(B)** Autoinhibited B-RAF:MEK1:14-3-3 complex (PDB ID: 6NYB (Park et al., 2019)). The CRD and the catalytic domains of B-RAF are shown in orange and violet, respectively. The position of the C-helix (shown in dark red) and the activation segment (shown in cyan) correspond to the autoinhibited state. **(C)** The Förster resonance energy transfer-based model of the complex between the FOXO4 Forkhead domain (FOXO4 DBD) and 14-3-3ζ (Silhan et al., 2009). The 14-3-3 binding motifs is located at the C-terminus of FOXO4-DBD. **(D)** The active B-RAF:MEK1:14-3-3 complex (PDB ID: 6Q0J (Park et al., 2019)). The 14-3-3 dimer anchors C-terminal 14-3-3 binding motifs of two B-RAF molecules, and the B-RAF kinase domains are oriented in the back-to-back fashion with the C-helix (shown in dark red) in a position consistent with the active conformation. The figure was prepared with PyMOL (https://pymol.org/2/).

The chaperon activity of 14-3-3 proteins is also involved in the regulation of *Pseudomonas* exotoxin-S and -T (ExoS and ExoT). Crystal structures of ExoS:14-3-3β and ExoT:14-3-3β complexes revealed extensive hydrophobic binding interfaces, which are sufficient for stable complex formation even in the absence of the amphipathic 14–3-3 binding LDLA-motif (Figure 4A) (Karlberg et al., 2018). Furthermore, 14-3-3 proteins protect ExoS against thermal aggregation, suggesting that 14-3-3 proteins activate ExoS and ExoT by protecting their hydrophobic surfaces from aggregation.

Another protein whose aggregation is prevented by 14-3-3 is α-synuclein. The accumulation of this neuronal protein is associated with Familial and idiopathic Parkinson disease (Plotegher et al., 2014). Conversely, 14-3-3 proteins stimulate the aggregation of unphosphorylated Tau protein, a neuronal protein involved in microtubule stabilization and a major component of intraneuronal neurofibrillary tangles in patients with Alzheimer disease (Sadik et al., 2009; Sluchanko and Gusev, 2011; Qureshi et al., 2013; Neves et al., 2021).

14-3-3-mediated regulation based on molecular interference also includes mechanisms in which 14-3-3 binding masks protein- or DNA-binding surfaces. For instance, phosducin (Pdc), a regulatory protein highly expressed in the retina and pineal gland, reduces signal amplification at the G protein level by binding to the G_t_βγ dimer and preventing its reassociation with G_t_α (Yoshida et al., 1994). Pdc binding to G_t_βγ is inhibited by phosphorylation of two serine residues within the N-terminal domain followed by binding to 14-3-3 proteins (Nakano et al., 2001; Thulin et al., 2001). Structural analysis of the Pdc:14-3-3ζ complex showed that 14-3-3 binding masks the G_t_βγ binding surface of Pdc, thereby blocking its interaction with G_t_βγ (Kacirova et al., 2015; Kacirova et al., 2017). 14-3-3-mediated physical occlusion of the protein-binding surface was also suggested for caspase-2, whose maturation from an inactive zymogen requires dimerization and autoproteolytic cleavage (Baliga et al., 2004). However, procaspase-2 phosphorylation at Ser^139^ and Ser^164^ induces its association with 14-3-3 proteins (Nutt et al., 2009), which blocks the dimerization interface of procaspase-2 (Kalabova et al., 2020).

Recently, several groups have reported cryo-EM and crystal structures of 14-3-3:B-RAF complexes, which provided detailed insights into the 14-3-3-dependent regulation of B-RAF kinase activity (Kondo et al., 2019; Park et al., 2019; Liau et al., 2020a; Liau et al., 2020b). 14-3-3 proteins regulate B-RAF both negatively and positively, and the 14-3-3 dimer inhibits B-RAF by simultaneous binding to two phosphorylated motifs (Ser^365^ and Ser^729^) that flank the kinase domain, as shown by the structure of the autoinhibited B-RAF:MEK1:14-3-3 complex. As a result, the cysteine rich domain of B-RAF is sequestered within the central channel of the 14-3-3 dimer, obstructing the dimerization interface of the B-RAF kinase domain (Figure 4B). Unable to dimerize, B-RAF remains in an inactive state, which prevents its Ras-driven activation and membrane recruitment (Park et al., 2019). Furthermore, recent molecular dynamics simulations have suggested that 14-3-3 binding also stabilizes the interactions of the Ras-binding (RBD) and cysteine-rich (CRD) domains with the catalytic domain of B-RAF, thereby blocking dimerization of the kinase domain (Zhang et al., 2021).

Molecular interference-based 14-3-3-mediated regulation also involves masking nucleic acid-binding surfaces, as described for the forkhead transcription factor FOXO4. As mentioned above, FOXO proteins contain two 14-3-3 binding motifs phosphorylated by Akt/PKB kinase, and 14-3-3 binding induces cytoplasmic sequestration of FOXOs by blocking their NLS (Brunet et al., 1999; Brunet et al., 2002; Obsilova et al., 2005). 14-3-3 binding motifs of FOXOs flank the DNA-binding Forkhead domain, with a second motif located at its C-terminus. Therefore, 14-3-3 dimer binding to this motif, in addition to interfering with the NLS, sequesters the Forkhead domain within the central channel of the 14-3-3 dimer and blocks its DNA-binding surface, thus inhibiting FOXO binding to the target DNA (Figure 4C) (Obsil et al., 2003; Silhan et al., 2009).

### 3.3 Facilitation of protein-protein interactions

14-3-3 protein dimerization enables scaffolding, which either stabilizes the oligomeric state of the target protein or anchors two different proteins near each other. A paradigmatic example of such a mechanism is the activation of plant plasma membrane H^+^-ATPase (Ottmann et al., 2007a). This enzyme is responsible for building up an electrochemical proton gradient across the plasma membrane, and its activity is triggered by phosphorylation of a threonine residue at the C-terminus and subsequent association with 14-3-3 proteins that stabilize the hexameric state of the H^+^-ATPase by bridging C-terminal regions of adjacent dimer subunits of ATPase.

As discussed above in Section 3.2.2, 14-3-3-mediated DAPK2 inhibition is, at least partly, also based on the stabilization of its dimers (Horvath et al., 2021). DAPK2 dimerization covers most of the kinase domain and part of the Ca^2+^/CaM-binding region and likely induces DAPK2 inhibition (Patel et al., 2011; Simon et al., 2016). SAXS-based structural analysis together with chemical cross-linking coupled to MS of the DAPK2: 14-3-3γ complex revealed that the 14-3-3γ dimer promotes DAPK2 dimerization by simultaneously binding to two DAPK2 molecules. Other factors that contribute to 14-3-3-mediated DAPK2 inhibition, namely protection of the DAPK2 inhibitory autophosphorylation site Ser^318^ from dephosphorylation and prevention of Ca^2+^/CaM binding, also help to stabilize the dimeric form of DAPK2.

Positive B-RAF regulation is another example of 14-3-3-mediated dimerization of the bound partner. B-RAF dimerization promotes catalytic activity and is the key event in RAF activation (Rajakulendran et al., 2009). 14-3-3-mediated B-RAF dimerization is triggered by B-RAF dephosphorylation at Ser^365^, which allows the formation of an active B-RAF dimer bound to the 14-3-3 dimer through Ser^729^-containing C-terminal segments of both B-RAF molecules. Within this complex, the B-RAF kinase domains are in the active conformation and oriented in a back-to-back fashion, as recently shown by the cryo-EM structure reported by Park et al. (Park et al., 2019) (Figure 4D). Moreover, molecular dynamics simulations suggest that B-RAF activation occurs through the interaction between the kinase domain of a 14-3-3-bound B-RAF molecule and the kinase domain of another B-RAF molecule without 14-3-3 (Zhang et al., 2021).

## 4 Conclusion

Recent high-resolution structures of 14-3-3 protein complexes with complete binding partners, such as *N*th1, HSPB6, ExoS, ExoT and B-RAF, together with studies based on hybrid approaches, have greatly expanded our understanding of mechanisms whereby 14-3-3 proteins regulate their binding partners. Many 14-3-3 complexes are involved in signaling pathways whose deregulation underlies various pathological conditions, including neurogenerative diseases, metabolic disorders and cancer. Therefore, targeted modulation of 14-3-3 PPIs is a promising strategy for treating these pathologies, as documented by several recent studies. Developing more effective and selective modulators of 14-3-3 PPIs requires, however, a more detailed understanding of the regulatory mechanisms, as well as high-resolution structures of the respective complexes. This knowledge will help to overcome the inherent selectivity problem of these compounds and to target not only the 14-3-3 phosphopeptide binding groove but also, and especially, other parts of the binding interfaces that are unique to specific complexes. Current advances in cryo-EM for structural analysis of multiprotein complexes, including 14-3-3 protein complexes, will inevitably increase the number of high-resolution structures of 14-3-3 complexes with complete binding partners. This structural information will allow us to elucidate further nuances of the role of these master regulators of cell signaling and to develop effective strategies for modulating their functions.
